# In quest of contact: phylogeography of helmeted terrapins (*Pelomedusa galeata*, *P. subrufa* sensu stricto)

**DOI:** 10.7717/peerj.4901

**Published:** 2018-06-05

**Authors:** Melita Vamberger, Margaretha D. Hofmeyr, Flora Ihlow, Uwe Fritz

**Affiliations:** 1 Museum of Zoology, Senckenberg Dresden, Dresden, Germany; 2 Department of Biodiversity and Conservation Biology, University of the Western Cape, Bellville, South Africa; 3 Zoological Research Museum Alexander Koenig, Bonn, Germany

**Keywords:** Africa, Biogeography, Climatic niche modeling, Namibia, South Africa, Taxonomy

## Abstract

Based on rangewide sampling and three mitochondrial and two nuclear markers (together up to 1,850 bp and 1,840 bp, respectively), we examine the phylogeography of two helmeted terrapin species (*Pelomedusa galeata* and *P. subrufa* sensu stricto) and infer shifts of climatically suitable spaces since the Last Glacial Maximum using a modeling approach. Whilst *P. galeata* displays significant phylogeographic structuring across its range and consists of two deeply divergent lineages that could represent distinct species, *P. subrufa* shows no obvious phylogeographic differentiation. This seems to be related to historically stable or fluctuating ranges. One of the lineages within *P. galeata* appears to be confined to the westernmost, winter-rainfall region of South Africa and deserves special conservational attention due to the scarcity of surface water. The other lineage is distributed further east and is differentiated in three weakly supported subclades with parapatric distribution; one occurring inland, and two along the south and east coasts, respectively. As far as is known, *P. subrufa* occurs in South Africa only in the northeast of the country (Limpopo, Mpumalanga) and we report the species for the first time from the Lapalala Wilderness Area in the Waterberg region (Limpopo), approximately 350 km further west than previously recorded. We confirmed the occurrence of *P. galeata* only 80 km south of Lapalala. Thus, a sympatric occurrence of *P. galeata* and* P. subrufa* is possible. Another putative contact zone, for the two lineages within *P. galeata*, must be located in the Western Cape region, and further contact zones are likely for the eastern subclades within *P. galeata*. The nuclear loci provided no evidence for gene flow across taxa or genetic clusters within taxa. Future investigations should use denser sampling from putative contact zones and more nuclear markers to re-examine this situation. Despite few phylogeographic studies published for southern African biota, it seems likely that differentiation follows general rules, and that climate and physiographic barriers (e.g., the Great Escarpment) have shaped phylogeographic patterns.

## Introduction

Helmeted terrapins of the genus *Pelomedusa* are widely distributed across sub-Saharan Africa, the southwestern Arabian Peninsula and Madagascar. Together with the African hinged terrapins of the genus *Pelusios*, helmeted terrapins constitute the family Pelomedusidae, a group of side-necked terrapins endemic to Africa (TTWG, 2017). *Pelomedusa* was long assumed to be monotypic, with the single species *P. subrufa* (Bonnaterre, 1789) sensu lato. However, recent research revealed *Pelomedusa* as one of the most speciose turtle genera of the world. Currently *Pelomedusa* contains 10 formally recognized species and a minimum of five unnamed candidate species (Vargas-Ramírez et al., 2010; Fritz et al., 2011; Fritz et al., 2014; Fritz et al., 2015; Vargas-Ramírez, Petzold & Fritz, 2016; Wong, Fong & Papenfuss, 2010; Petzold et al., 2014; Nagy et al., 2015).

Two *Pelomedusa* species have been recorded in South Africa (Petzold et al., 2014; Fritz et al., 2015). Most of the country is inhabited by *P. galeata* (Schoepff, 1792), which consists of two deeply divergent mitochondrial lineages. Each lineage can be considered as an unconfirmed candidate species sensu Padial et al. (2010), with pronounced genetic divergences resembling those of distinct turtle species (Petzold et al., 2014; Fritz et al., 2015). One of these candidate species, lineage I, is widely distributed across the central and eastern provinces of South Africa (Eastern Cape, Free State, Gauteng, KwaZulu-Natal, North West and eastern parts of the Northern and Western Cape). The other candidate species, lineage II, appears to be confined to the westernmost part of the country in the Western and Northern Cape Provinces (Petzold et al., 2014). In addition to the two lineages of *P. galeata*, another species (*P. subrufa* sensu stricto) has been recorded from the Kruger National Park region in the northeast of South Africa (Limpopo and Mpumalanga; Petzold et al., 2014; Fritz et al., 2015).

*Pelomedusa subrufa* sensu stricto is distributed from southern Angola and Namibia through Botswana and the Democratic Republic of the Congo to Tanzania and Malawi (Petzold et al., 2014). Most likely, it occurs also in Zambia and Mozambique. Introduced populations live on Madagascar (Vargas-Ramírez et al., 2010; Wong, Fong & Papenfuss, 2010; Petzold et al., 2014). *Pelomedusa galeata* and *P. subrufa* are sister taxa and constitute together the sister clade to the remaining *Pelomedusa* species from more northerly regions of Africa and the Arabian Peninsula (Vargas-Ramírez et al., 2010; Fritz et al., 2011; Petzold et al., 2014). Even though a considerable number of mitochondrial DNA (mtDNA) sequences have been published by previous studies (Vargas-Ramírez et al., 2010; Vargas-Ramírez, Petzold & Fritz, 2016; Wong, Fong & Papenfuss, 2010; Petzold et al., 2014; Fritz et al., 2015), phylogeographic structuring within *P. galeata* and *P. subrufa* has not yet been examined.

For the present study, we expanded the previous sampling considerably and collected 43 additional *Pelomedusa* samples to (1) investigate phylogeographic structure of the two species and (2) delimit the distributions of *P. subrufa* and the *P. galeata* lineages across South Africa. In doing so, we used the same three mitochondrial markers (together up to 1,850 bp) as in our previous studies and sequenced for crucial samples two nuclear loci (up to 1,840 bp). In addition, using genetically verified records, we calculated climatic niche models for each species and genetic lineage.

## Materials and Methods

### Sampling and laboratory procedures

Fieldwork and sampling in South Africa were permitted by the Limpopo Provincial Government (permit ZA/Lp/80202), Ezemvelo KwaZulu-Natal Wildlife (permit OP 139/2017), CapeNature (permit AAA007-00212-0056), the Department of Environmental Affairs, Eastern Cape (permit CRO117/13C & CRO 118/13CR), and Biodiversity Northern Cape Province (permit 245/2015). Fieldwork and sampling in Namibia were permitted by the Ministry of Environment and Tourism (permit 1910/2014). Terrapins were hand-collected or captured using baited traps, and blood and saliva samples were taken as approved by the Ethics Committee of the University of the Western Cape under ethical clearance number ScRiRC2008/39. Terrapins were released after sampling at the capture sites.

Using alcohol-preserved blood or saliva samples and wet laboratory approaches for fresh material as described in Fritz et al. (2014), we generated sequences of the partial 12S rRNA gene (360 bp) for 36 *Pelomedusa galeata* and seven *P. subrufa* sensu stricto from South Africa and Namibia. Another sequenced mtDNA fragment of these samples comprised the partial ND4 gene plus adjacent DNA coding for tRNAs (816 bp), and a third mtDNA fragment corresponded to the partial cytochrome *b* (cyt *b*) gene (674 bp). In addition, we used homologous mtDNA sequences of helmeted terrapins from other investigations (Vargas-Ramírez et al., 2010; Wong, Fong & Papenfuss, 2010; Fritz et al., 2011; Fritz et al., 2014; Fritz et al., 2015; Petzold et al., 2014; Nagy et al., 2015). Including previously published material, we studied mtDNA sequences of 116 *P. galeata* and 41 *P. subrufa* sensu stricto and georeferenced their collection sites (Table S1).

In addition, we sequenced two nuclear loci of samples representing all mitochondrial lineages and subclades and almost all sampling sites (Table S1). One locus, the intron 1 of the RNA fingerprint protein 35 gene (R35) has been previously shown to be species-diagnostic for *P. galeata* and *P. subrufa* (Vargas-Ramírez et al., 2010). The other locus, including coding and non-coding parts of the ornithine decarboxylase gene (ODC), is relatively variable in chelonians (Fritz et al., 2012; Praschag et al., 2017) and therefore looked promising to be also species-specific. Laboratory procedures for the nuclear loci followed Praschag et al. (2017) except that we applied newly designed internal primers for sequencing the R35 gene (forward: GCAAGGAAAAATGTTTG, reverse: ACGCTGACTCCATGCACA). The resulting R35 sequences were 1,101 bp long. The ODC sequences comprised a hardly readable simple-sequence-repeat (SSR) region, which could not be sequenced for all samples. We excluded this region from further analyses, yielding 739 bp length. Including some previously published data (Vargas-Ramírez et al., 2010), R35 sequences were available for 37 *P. galeata* and 16 *P. subrufa*. For the ODC gene, sequences were available for 31 *P. galeata* and 10 *P. subrufa*.

### Phylogenetic analyses and uncorrected *p* distances of mtDNA

We concatenated individual mtDNA fragments for phylogenetic analyses and merged this dataset with previously published sequences, resulting in an alignment of 1,850 bp length that included 233 *Pelomedusa* sequences (also including sites outside South Africa and Namibia, and other species). *Pelusios sinuatus* served as the outgroup. For *Pelomedusa galeata*, *P. subrufa* and the outgroup, accession numbers and collection sites are given in Table S1; for other species, see Petzold et al. (2014), Fritz et al. (2015), and Nagy et al. (2015). We assessed the best partitioning scheme using PARTITIONFINDER (Lanfear et al., 2012) and the Bayesian Information Criterion (BIC). Accordingly, we partitioned the dataset using each codon position of the protein-coding genes, the 12S gene and the lumped DNA coding for tRNAs as a distinct partition. We inferred phylogenetic relationships under Maximum Likelihood using RAxML 7.2.8 (Stamatakis, 2006) and the GTR + G substitution model across all partitions. We performed five independent ML searches using different starting conditions and the fast bootstrap algorithm to explore the robustness of the results by comparing the best trees. Then, we calculated 1,000 non-parametric thorough bootstrap replicates and plotted the values against the best tree. In addition, we calculated uncorrected *p* distances for each mtDNA fragment using MEGA 7.0.21 (Kumar, Stecher & Tamura, 2016) and the pairwise deletion option.

### Parsimony networks

We calculated for concatenated mitochondrial sequences a parsimony network using POPART (http://popart.otago.ac.nz). Since the underlying algorithm is sensitive to missing data, we excluded all individuals with lacking genes. In addition, we removed all individual missing sites and homologous data, resulting in an alignment of 1,602 bp length, which contained 87 sequences of *P. galeata* and 18 sequences of *P. subrufa*.

For network construction of nuclear data, we phased heterozygous R35 and ODC sequences using the PHASE algorithm in DNASP 5.10 (Librado & Rozas, 2009). For R35, we built two networks because the sequences of Vargas-Ramírez et al. (2010) were approximately 300 bp shorter than ours. One network comprised only our 86 phased sequences of 1,101 bp length, whereas the second also contained the previously published data (in total 106 phased sequences). It was based on an alignment trimmed to 699 bp to match the sequence lengths of Vargas-Ramírez et al. (2010).

### Climatic niche models

To assess whether historical climate fluctuations influenced the distribution of *Pelomedusa galeata* and *P. subrufa*, we computed climatic niche models for present conditions as well as for the Last Glacial Maximum (LGM) and the mid-Holocene using the machine-learning algorithm MAXENT 3.3.3k (Phillips, Anderson & Schapire, 2006; Phillips & Dudík, 2008). To remove spatial autocorrelation, we filtered the genetically verified occurrences for each species, as well as for subclades Ia-Ic and lineage II of *P. galeata*, retaining only one record per sampling site, and supplemented the dataset with unambiguously assignable records from VertNet (http://vertnet.org/). The resulting datasets contained 31 localities for *P. subrufa* (23 own and eight VertNet records) and 51 for *P. galeata* (47 own and four VertNet records). Of the latter, 14 localities corresponded to subclade Ia, 10 to Ib, 17 to Ic, and 10 to lineage II.

We obtained eight uncorrelated bioclimatic predictors (*R*_2_ < 0.75) with a spatial resolution of 2.5 arc minutes (∼5 km at the equator) from WorldClim (http://www.worldclim.org/) for current and past climatic conditions (mid-Holocene, ∼6,000 BP; LGM, ∼21,000 BP). For the mid-Holocene and LGM, we used datasets for three different general circulation model scenarios, namely the Community Climate System Model (CCSM4), the Model for Interdisciplinary Research on Climate (MIROC-ESM), and the Max-Planck-Institute Earth System Model P (MPI-ESM-P) that were statistically downscaled to a spatial resolution of 2.5 arc min. The following predictor variables were selected and clipped to a rectangular study extent: Bio 3 = isothermality (Bio2/Bio7)(*100), Bio 5 = maximum temperature of warmest month, Bio 7 = temperature annual range, Bio 8 = mean temperature of the wettest quarter, Bio 9 = mean temperature of the driest quarter, Bio 15 = precipitation of the wettest quarter, Bio 17 = precipitation of the driest month, and Bio 18 = precipitation of the warmest quarter.

We trained models separately for *P. subrufa*, *P. galeata*, and for subclades Ia-Ic and lineage II of *P. galeata* using circular buffers of 200 km surrounding the respective records and projected the results onto the full study extent for current and past conditions. In MAXENT, we selected linear, quadratic and hinge features to reduce model complexity and applied a bootstrapping approach with 100 replicates randomly splitting the records into 80% used for training and 20% for model evaluation. We performed a maximum number of 5,000 iterations and used the area under the curve AUC (Swets, 1988) for model evaluation. We used the average projection across 100 replicates for further processing, wherein we applied the “minimum training presence logistic threshold” as presence-absence threshold.

## Results

### Phylogenetic analyses of mtDNA

**Figure 1:**
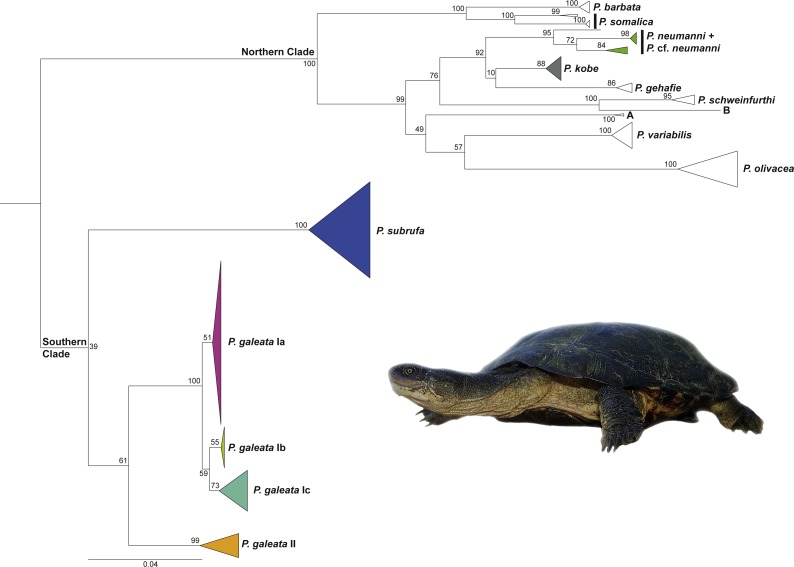
Maximum Likelihood tree for *Pelomedusa* using 1,850 bp of mtDNA with bootstrap values indicated. Terminal clades collapsed to cartoons; colors of cartoons correspond to Fig. 2 (open cartoons represent clades beyond the map sector). The northern clade contains several distinct candidate species (candidate species A and B; deeply divergent clades within species; see Petzold et al., 2014; Fritz et al., 2015; Nagy et al., 2015). Outgroup (*Pelusios sinuatus*) removed for clarity. For the complete tree displaying individual samples, see Fig. S1. Inset: *Pelomedusa galeata* (subclade Ib), Nederland Farm, KwaZulu-Natal. Photo credit: M. Vamberger.

The general branching pattern of our phylogenetic tree (Fig. 1) was in agreement with previous publications (Vargas-Ramírez et al., 2010; Wong, Fong & Papenfuss, 2010; Fritz et al., 2014; Fritz et al., 2015; Petzold et al., 2014) in that a well-supported major clade included all species and candidate species from the northern part of the distribution range of the genus *Pelomedusa*. This northern clade was sister to a weakly supported southern clade comprising *Pelomedusa galeata* and *P. subrufa* sensu stricto. Whilst the monophyly of *P. subrufa* was well supported, the monophyly of *P. galeata* received only weak bootstrap support of 61.

**Figure 2:**
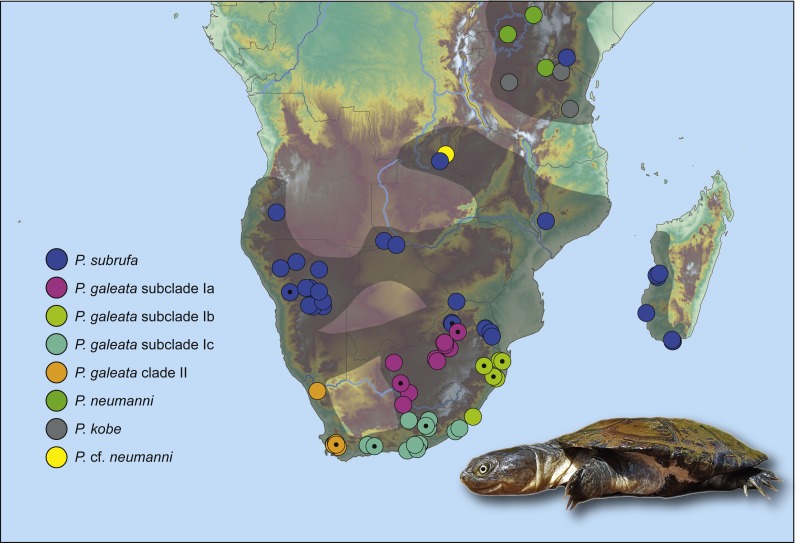
Genetically verified records of *Pelomedusa galeata*, *P. subrufa* sensu stricto and geographically neighboring species in southern Africa and adjacent regions. Records are based on the present study and Petzold et al. (2014), Fritz et al. (2015) and Nagy et al. (2015); for details, see Table S1. Symbols with central black circles represent new records. For localities of samples with nuclear DNA data, see Fig. S2. Range of *Pelomedusa* spp. shaded (combined from Bates et al., 2014; TTWG, 2017). Inset: *Pelomedusa subrufa*, Lapalala Wilderness Area, Limpopo. Photo credit: F. Ihlow.

*Pelomedusa galeata* showed clear genetic structuring, with two well-supported clades (clade I and clade II) corresponding to deeply divergent mitochondrial lineages. One of these mitochondrial lineages (clade I) comprised three weakly supported subclades; Ia, Ib, and Ic. Subclade Ia was sister to a weakly supported, more inclusive clade with subclades Ib and Ic. Localities for subclade Ia were from the interior of South Africa, at high elevations above the Great Escarpment, where summer-rainfall prevails (Fig. 2; records in the provinces of the Free State, Gauteng, Limpopo, North West, Northern Cape). Samples in subclade Ib approximated geographically to the subtropical (low-elevation) summer-rainfall region along the northeast coast of South Africa (Eastern Cape, KwaZulu-Natal). The samples of subclade Ic were from the south coast and adjacent inland regions, mostly below the Great Escarpment, with all-year (aseasonal) rain (Eastern Cape, Western Cape). The other mitochondrial lineage (clade II) was from the western, winter-rainfall region of South Africa, represented by several samples from the southwestern Cape and the historical type specimen of *Pelomedusa galeata devilliersi* Hewitt (1935) from a site in the arid northwest of South Africa, close to the Namibian border. Even though *P. subrufa* also showed sequence variation, there was no obvious geographic pattern (Fig. 1 and Fig. S1). Considering the wide distribution of *P. subrufa* (Fig. 2), this is unexpected and contrasts with the phylogeographic structure found in *P. galeata*. Our new records for *P. subrufa* from the Lapalala Wilderness Area in the Waterberg region (Limpopo) currently represent the westernmost known occurrences for this species in South Africa. Before it was only known from the western border region of the Kruger Park.

### Uncorrected *p* distances of mtDNA

Mean uncorrected sequence divergences between *Pelomedusa galeata* and *P. subrufa* amounted to 5.6% for the 12S fragment and 10.3% for the cyt *b* fragment, two genes that have previously been used for species delimitation in *Pelomedusa* (Petzold et al., 2014). Within all *P. galeata* (lineages I and II together) and within *P. subrufa*, the sequence divergence for 12S was 0.7% for each. For cyt *b*, the divergences were 2.0% for *P. galeata* and 1.3% for *P. subrufa* (Table 1). Between lineages I and II of *P. galeata*, the sequence divergence was 2.2% for 12S and 7.1% for cyt *b*. Additional values, and divergences for the mtDNA fragment comprised of the ND4 and tRNA genes, are summarized in Table 1.

**Table 1:** Average uncorrected *p* distances (percentages) for the three studied mtDNA fragments. Between-group divergences below diagonal; within-group divergences in bold on the diagonal.

12S	*gal* (all)	*gal* I (all)	*gal* II	*gal* Ia	*gal* Ib	*gal* Ic	*sub*
*galeata* (all)	**0.7**						
*galeata* I (all)	–	**0.4**					
*galeata* II	–	2.2	**0.3**				
*galeata* Ia	–	–	2.1	**0**			
*galeata* Ib	–	–	2.2	0.4	**0.1**		
*galeata* Ic	–	–	2.7	1.0	0.7	**0.2**	
*subrufa*	5.6	5.7	4.8	5.6	5.7	6.1	**0.7**

cyt *b*	*gal* (all)	*gal* I (all)	*gal* II	*gal* Ia	*gal* Ib	*gal* Ic	*sub*
*galeata* (all)	**2.0**						
*galeata* I (all)	–	**0.8**					
*galeata* II	–	7.1	**0.5**				
*galeata* Ia	–	–	6.8	**0**			
*galeata* Ib	–	–	7.6	1.7	**0.1**		
*galeata* Ic	–	–	7.5	1.2	1.6	**0.3**	
*subrufa*	10.3	10.1	12.1	10.0	10.1	10.4	**1.3**

ND4 + tRNAs	*gal* (all)	*gal* I (all)	*gal* II	*gal* Ia	*gal* Ib	*gal* Ic	*sub*
*galeata* (all)	**1.5**						
*galeata* I (all)	–	**0.6**					
*galeata* II	–	5.0	**0.6**				
*galeata* Ia	–	–	5.0	**0.1**			
*galeata* Ib	–	–	5.0	1.0	**0.1**		
*galeata* Ic	–	–	5.0	1.3	1.1	**0.5**	
*subrufa*	7.1	7.1	7.6	7.0	7.2	7.1	**0.8**

### Parsimony networks

**Figure 3:**
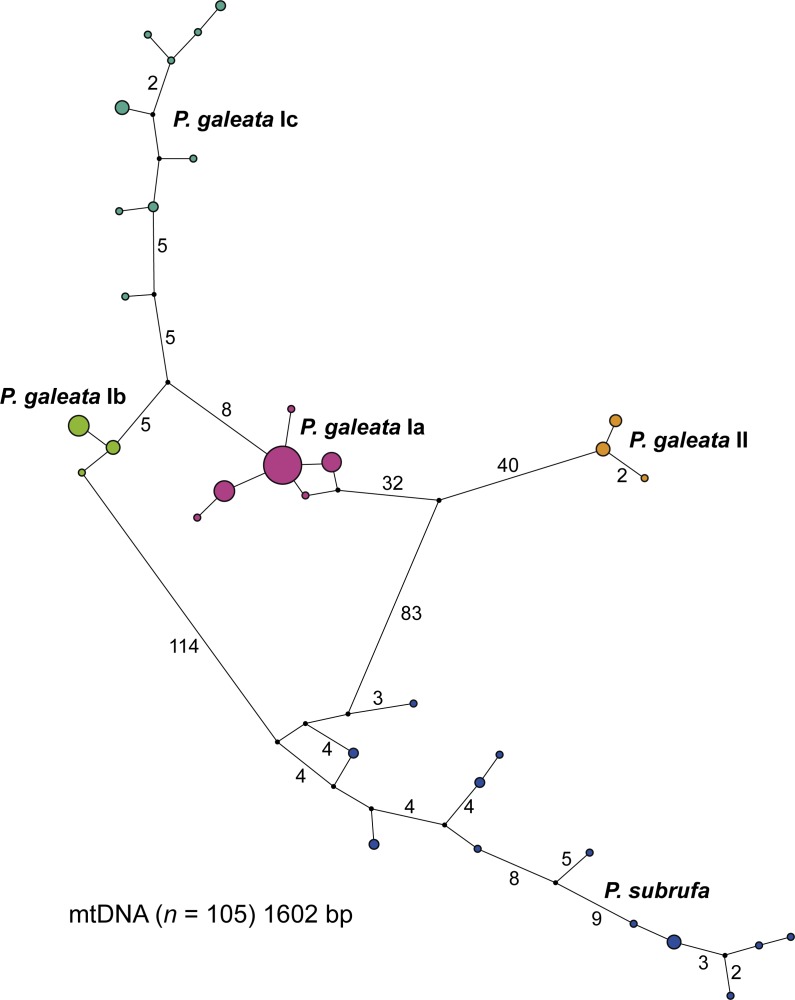
Parsimony network for mitochondrial haplotypes of *Pelomedusa galeata* and *P. subrufa.* Symbol size corresponds to haplotype frequency; haplotypes colored according to mitochondrial lineages (Figs. 1 and 2). Lines connecting haplotypes represent one mutational step except when otherwise indicated by numbers. Missing node haplotypes, small black circles.

The concatenated mitochondrial sequences were grouped in five distinct clusters (Fig. 3). Among the four clusters corresponding to the clades and subclades of *Pelomedusa galeata*, a maximum of 100 mutation steps occurred; among the haplotypes of *P. subrufa*, a maximum of 38 steps. Clade II of *P. galeata* was separated from the most similar haplotype of clade I (subclade Ia) by a minimum of 73 steps; subclade Ia differed from subclade Ib by a minimum of 13 steps, and from subclade Ic by 14 steps. Subclades Ib and Ic diverged by a minimum of 11 steps from one another. The haplotype clusters of *P. galeata* and *P. subrufa* were connected in a loop, with subclades Ia and Ib differing from *P. subrufa* by a minimum of 119 mutation steps. Clade II of *P. galeata* was separated by a minimum of 126 mutations from *P. subrufa*.

**Figure 4:**
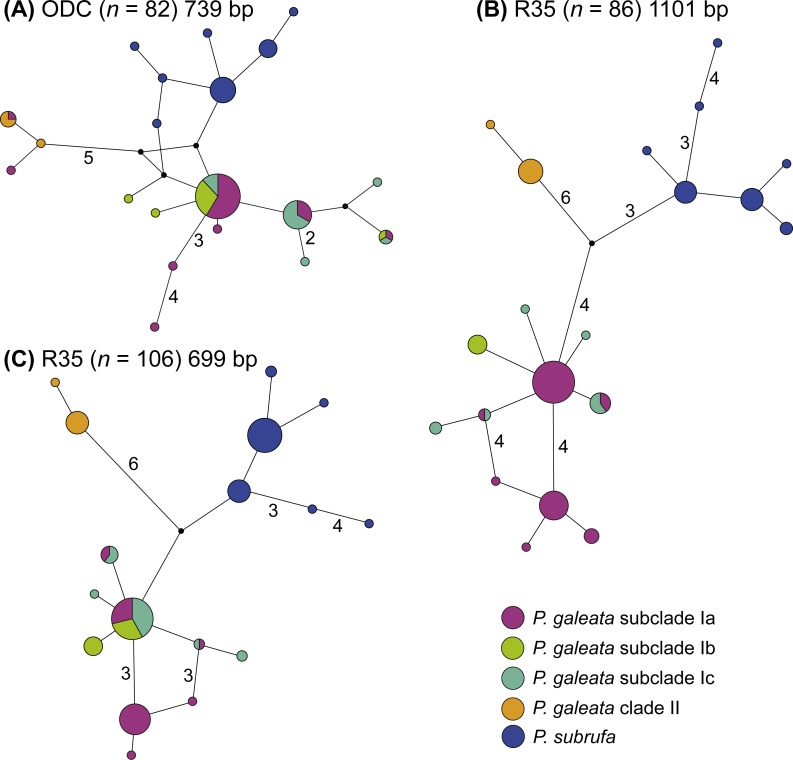
Parsimony networks for nuclear haplotypes of *Pelomedusa galeata* and *P. subrufa* (A–C). Symbol size corresponds to haplotype frequency; haplotypes colored according to mitochondrial lineages (Figs. 1 and 2). Lines connecting haplotypes represent one mutational step except when otherwise indicated by numbers. Missing node haplotypes, small black circles. For geographic distribution of shared haplotypes, see Fig. S3.

Even though much less variation occurred in the nuclear data, no haplotype sharing was observed between *P. galeata* and *P. subrufa* (Fig. 4). In the ODC network, haplotypes of *P. galeata* differed by a minimum of two mutations from haplotypes of *P. subrufa*. In the R35 network comprising the long sequences (1,101 bp), the two species differed by a minimum of seven mutations; in the R35 network with the short sequences (699 bp), the minimum was two steps. For the samples corresponding to the mitochondrial lineages within *P. galeata*, haplotype sharing was observed for the ODC gene between subclades Ia and clade II, and between subclades Ia, Ib, and Ic. However, unique haplotypes occurred in each subclade. Both R35 networks consisted of three haplotype clusters, one corresponding to *P. subrufa*, and the two others to lineages I and II of *P. galeata*, respectively. No haplotypes were shared between lineage I and lineage II. The number of mutations separating the two lineages of *P. galeata* resembled (1,101 bp network) or clearly exceeded (699 bp network) the divergence between *P. subrufa* and the two haplotype clusters of *P. galeata*. Haplotype sharing was observed for subclades Ia, Ib, and Ic of *P. galeata*, but to a lesser extent in the network based on the longer sequences. Unique haplotypes occurred for each subclade. The occurrence of shared haplotypes of the two lineages or the individual subclades of *P. galeata* showed no correlation with geography (Fig. S3), rather suggestive of ancestral polymorphism than of gene flow.

**Figure 5:**
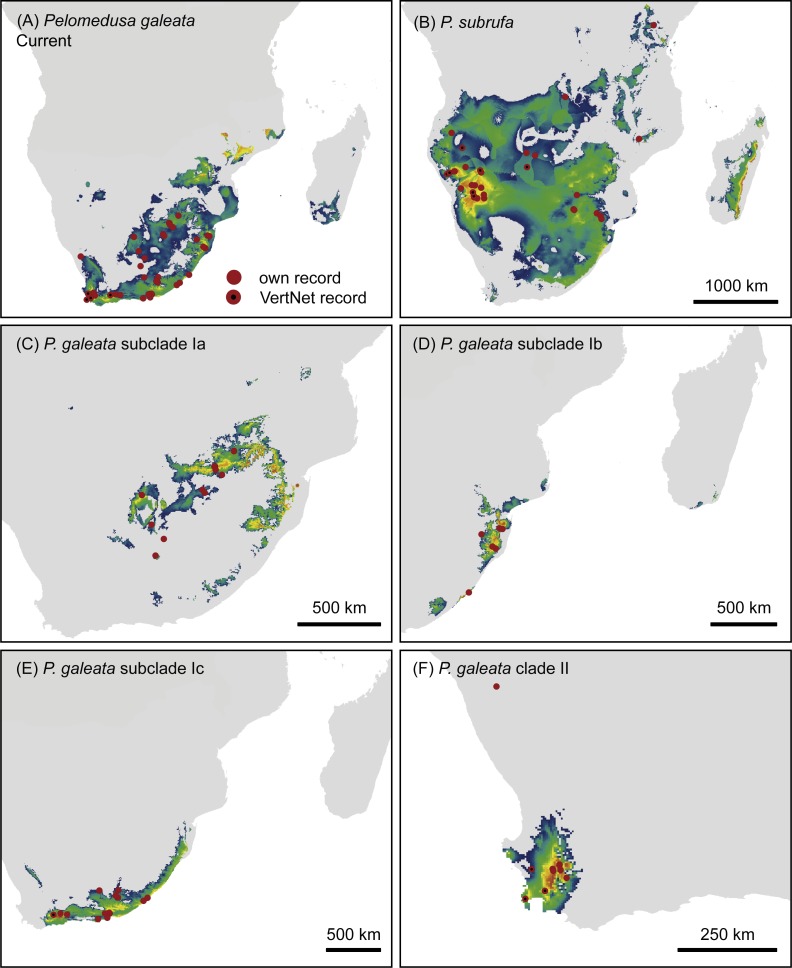
Climatic niche models for *Pelomedusa galeata* (A), *P. subrufa* (B) and genetic clusters within *P. galeata* (C–F) under current climatic conditions. Probabilities for habitat suitability range from low (blue) to high (orange).

### Climatic niche models

The models for current climatic conditions revealed distinct areas of climatically suitable space for the two *Pelomedusa species* (Fig. 5), with some overlap in north-eastern South Africa. The discrimination capability, expressed as AUC_test_ scores, of both models was high (*P. subrufa* = 0.79, *P. galeata* = 0.73), indicating a good discrimination of suitable and unsuitable areas. Across all 100 replicates, contributions of variables beyond 20% showed both models to be strongly affected by temperature and precipitation during the driest month and quarter (Table 2: Bio 9, Bio 17). However, the model for the widespread *P. subrufa* was also influenced by temperature during the wettest quarter (Bio 8 > 10%), whilst *P. galeata* was revealed to be strongly dependent on temperature annual range (Bio 7 >20%) and precipitation during the warmest quarter (Bio 18 > 10%), two variables less important for *P. subrufa*. Potentially suitable areas for the subclades of *P. galeata* were highly distinct (Fig. 5) and described by different predictors. For subclade Ia, inhabiting a highland summer-rainfall area, temperature annual range and mean temperature of the wettest and driest quarter contributed the most (Bio 7 and Bio 8 > 20%, Bio 9∼20%). However, the model was also impacted by precipitation during the driest month (Bio 17 > 10%). For subclade Ib, inhabiting a subtropical low elevation summer-rainfall area, the model was exclusively shaped by temperature-related variables (Bio 7∼40%; Bio 3 and Bio 9 >20%), whilst precipitation-related predictors contributed less than 5%. For subclade Ic, inhabiting the southern coast and adjacent inland regions characterized by aseasonal rainfall, variable contribution was highest for precipitation during the driest month (Bio 17 = 36%), precipitation seasonality, and temperature during the wettest quarter (Bio 15, Bio 8 > 10%). For subclade II from the western winter-rainfall area of South Africa, precipitation of the wettest quarter was the variable with the highest importance (Bio 16∼77%), whilst no other predictor exceeded 10%. All models for the subclades of *P. galeata* received excellent AUC_test_ scores (Ia = 0.81, Ib = 0.75, Ic = 0.78, II = 0.93) and suggest mutually exclusive climatic niches.

**Table 2:** Contribution of selected bioclimatic predictor variables to current predictions as derived from MAXENT. Values exceeding 10% in bold.

ID	Variable contribution % current					
	*P. subrufa*	*P. galeata*	Ia	Ib	Ic	II
Bio 3	7.6	1.8	1.8	**15.0**	9.7	2.4
Bio 5	9.2	9.6	0.7	5.9	0.6	6.7
Bio 7	7.5	**21.4**	**24.5**	**39.2**	9.4	2.0
Bio 8	**10.4**	7.9	**25.1**	9.5	**14.3**	1.5
Bio 9	**24.9**	**20.6**	**19.6**	**17.5**	4.9	3.7
Bio 15	2.2	1.6	2.9	4.9	**16.3**	5.2
Bio 16	9.5	8.0	9.4	2.8	5.8	**77.4**
Bio 17	**21.4**	**19.1**	**10.6**	4.6	**36.6**	1.0
Bio 18	7.3	**10.0**	5.4	0.6	2.4	0.1

**Figure 6:**
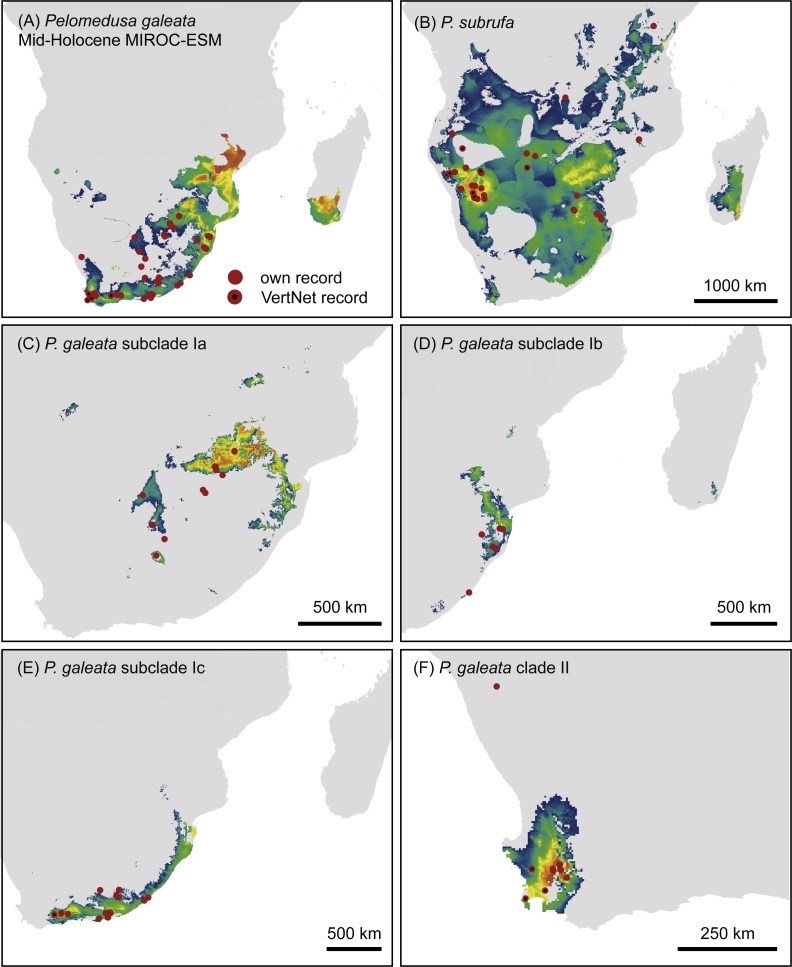
Predicted climatic niches for *Pelomedusa galeata* (A), *P. subrufa* (B) and genetic clusters within *P. galeata* (C–F) during the mid-Holocene (MIROC-ESM). Probabilities for habitat suitability range from low (blue) to high (orange). For CCSM4 and MPI-ESM-P models, see Figs. S4–S7.

**Figure 7:**
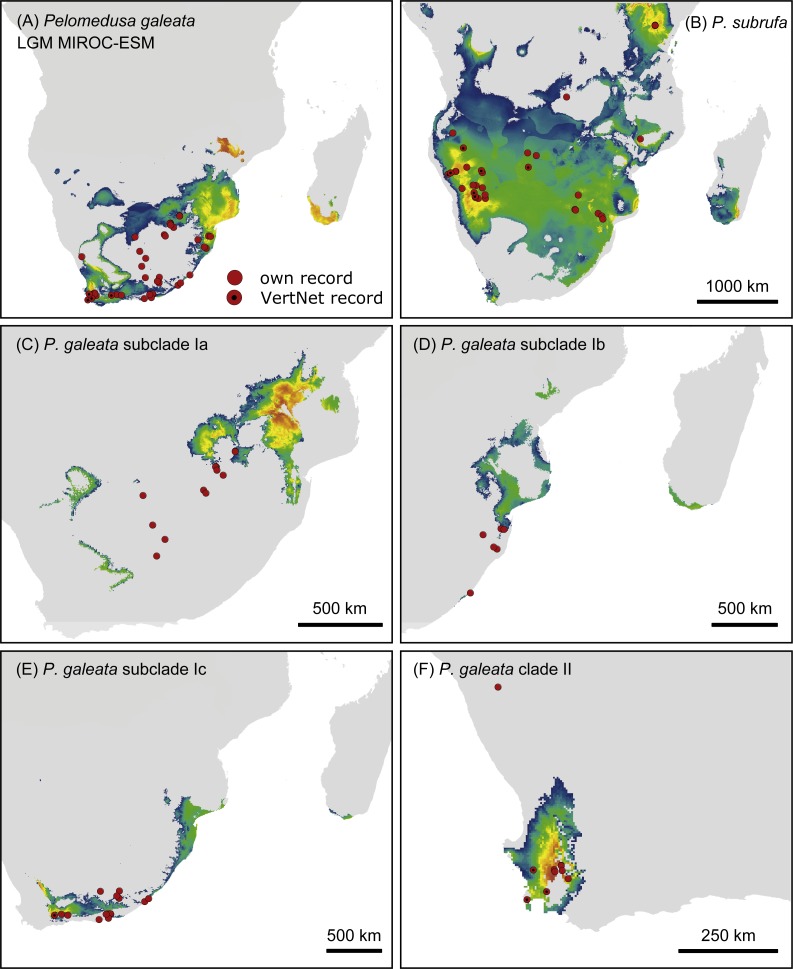
Predicted climatic niches for *Pelomedusa galeata* (A), *P. subrufa* (B) and genetic clusters within *P. galeata* (C–F) during the Last Glacial Maximum (MIROC-ESM). Probabilities for habitat suitability range from low (blue) to high (orange). For CCSM4 and MPI-ESM-P models, see Figs. S4–S7.

Projections onto past climatic conditions found similar patterns across all three scenarios (Figs. 6 and 7, Figs. S4–S7) with high discrimination abilities (MIROC-ESM: *P. subrufa* = 0.78, *P. galeata* = 0.73, Ia = 0.84, Ib = 0.77, Ic = 0.78, II = 0.95; CCSM4: *P. subrufa* = 0.73, *P. galeata* = 0.80, Ia = 0.86, Ib = 0.77, Ic = 0.80, II = 0.91; MPI-ESM-P: *P. subrufa* = 0.71, *P. galeata* = 0.80, Ia = 0.85, Ib = 0.81, Ic = 0.80, II = 0.91).

Projections onto climatic conditions of the LGM revealed for *P. subrufa* a suitable area resembling the present situation, whilst the space for *P. galeata* shifted considerably, excluding a large area in the center of the extant distribution range. When the models for subclades Ia-Ic and lineage II of *P. galeata* were inspected individually, the models for subclade Ic and lineage II matched well with extant conditions. However, the models for subclades Ia and Ib shifted to the northeast, outside the current niche, but remained geographically mutually exclusive (Figs. 6 and 7). All projections onto LGM conditions yielded high AUC_test_ scores (MIROC-ESM: *P. subrufa* = 0.80, *P. galeata* = 0.71, Ia = 0.85, Ib = 0.77, Ic = 0.76, II = 0.91; CCSM4: *P. subrufa* = 0.77, *P. galeata* = 0.73, Ia = 0.83, Ib = 0.77, Ic = 0.78, II = 0.94; MPI-ESM-P: *P. subrufa* = 0.79, *P. galeata* = 0.72, Ia = 0.85, Ib = 0.77, Ic = 0.76, II = 0.94).

## Discussion

According to our present investigation, *Pelomedusa galeata* and *P. subrufa* sensu stricto, as well as the genetic lineages and subclades within *P. galeata*, occur parapatrically. In South Africa, *P. subrufa* is restricted to the northeast of the country, but with a wider distribution ranging approximately 350 km further westward than previously known (Fritz et al., 2015). All known South African records for *P. subrufa* lie in Mpumalanga and Limpopo. Our new records from the Lapalala Wilderness Area (Limpopo) represent the westernmost localities in South Africa. These sites are only 80 km north of the nearest record of *P. galeata* (subclade Ia) at Mosdene Farm, Mookgopong (Limpopo). Thus, a sympatric occurrence of the two species seems possible, as first suggested by Vargas-Ramírez, Petzold & Fritz (2016) using a species distribution modeling approach and corroborated by the modeling results of the present study. Our results indicate an even wider area of potential overlap, embracing the central and northeastern parts of South Africa (Fig. 5).

Our study confirms that the taxon currently identified as *P. galeata* is composed of two deeply divergent genetic lineages (I and II; Figs. 1 and 3), as earlier revealed by Petzold et al. (2014) using mtDNA data. In addition, we found that lineage I is differentiated in three weakly supported subclades (Ia-Ic). Our nuclear DNA data also confirmed the distinctness of lineages I and II (Fig. 4). We found lineage II restricted to westernmost South Africa, with several new records in the southwestern part of the Western Cape (Fig. 2; Table S1). Our climatic niche modeling supports that lineage II occurs only in the winter-rainfall region of South Africa. Besides the localities in the southwestern Western Cape, there is a single record in the Northern Cape. It corresponds to the collection site of the historical type specimen of *Pelomedusa galeata devilliersi* (Hewitt, 1935), a taxon considered to be synonymous with *P. galeata* (Fritz et al., 2014; Petzold et al., 2014). However, the mtDNA sequences of the type specimen (Fritz et al., 2014) differ from those of the southwestern terrapins. Moreover, the collection site of the type lies far beyond the predicted range of lineage II (Fig. 5). This situation requires further research, including fieldwork around the type locality of *P. g. devilliersi*, to confirm the occurrence of *Pelomedusa* there and to obtain fresh material.

*Pelomedusa* is generally rare in northwestern South Africa, with a large distributional gap between the western and central localities (Fig. 2). Western South Africa displays a south to north aridity gradient, which may play a role for the genetic differentiation of several *other* reptile species, for example, *Chersina angulata* (Daniels et al., 2007), *Trachylepis sulcata* (Portik, Bauer & Jackman, 2011), and *Bitis arietans* (Barlow et al., 2013).

The phylogeographic pattern of *P. galeata* roughly parallels that of the puff adder *B. arietans*, as described in Barlow et al. (2013). The westerly clade of *B. arietans* corresponds to lineage II of *P. galeata*, the southern-to-eastern coastal clade to subclade Ic, the northeastern subclade to subclade Ib, and the northwestern subclade to subclade Ia. Similar to our conclusions for *Pelomedusa* (see below), Barlow et al. (2013) ascribed the phylogeographic pattern of *B. arietans* to climatic oscillations during the Plio-Pleistocene, with populations retracting to coastal and northern refugia when interior regions became inhospitable during glacial maxima. These authors proposed that low temperatures in the interior rather than aridity made the habitat inhospitable. It is noteworthy for *Pelomedusa*, but also for clawed frogs (*Xenopus laevis*) living in similar habitats (Furman et al., 2015) and for *B. arietans* (Barlow et al., 2013) living in very different habitats, that phylogeographic breaks coincide in South Africa with the Great Escarpment, i.e., with lineages confined either to lowland or highland. This suggests that physiographic structures shaped in concert with climatic factors the current phylogeographic structures of South African biota. Furthermore, the phylogeographic similarities of very different taxa imply that general paradigms exist, like in the Western Palearctic or the Nearctic (Hewitt, 2000; Hewitt, 2011; Schmitt, 2007; Schmitt & Varga, 2012; Riddle, 2016). However, the understanding of the phylogeography of sub-Saharan biota is distinctly less advanced than for the Western Palearctic and the Nearctic (Hewitt, 2000; Hewitt, 2011; Schmitt, 2007), even in comparatively well-researched countries like South Africa and Namibia. For unveiling general differentiation patterns and understanding their causes, the description of species-specific phylogeographic patterns is the necessary prerequisite. This study contributes to this ultimate goal and presents basic phylogeographic data for two wide-ranging terrapin species from southern Africa.

As expected for thermophilic semiaquatic species like terrapins, our niche modeling confirms that the phylogeographic structure of *Pelomedusa* has been significantly impacted by temperature and aridity. Yet, *P. galeata* has been much more affected by climatic fluctuations since the LGM than *P. subrufa*. This difference could explain the absence of phylogeographic structuring in *P. subrufa* (Fig. 1 and Fig. S1). Moreover, our models suggested for each of the four genetic clusters within *P. galeata* a distinct climatic niche (Fig. 5: subclades Ia-Ic, lineage II). Potentially suitable spaces for subclades Ia and Ib shifted northeastward during the LGM and moved southwestward in the Holocene (Figs. 5–7 and Figs. S4–S7). Since climatic instability and repeated shifts of grassland and semidesert biomes have been inferred for the past 140,000 years for the concerned regions (Huntley et al., 2016), it is likely that part of the range of *P. galeata* was highly dynamic for a long time, a situation contributing to phylogeographic divergence. In contrast, our models propose that the distributions of *P. subrufa* and of subclade Ic and lineage II of *P. galeata* remained largely stable since the LGM.

The present study also has taxonomic and conservational implications. The genetic distances between clade I and clade II of *P. galeata* (Table 1), with 2.2% mean divergence for the 12S gene and 7.1% for the cyt *b* gene, resemble divergences between currently recognized *Pelomedusa* species (2.6% to 12.2% for 12S, 5.6% to 18.6% for cyt *b*; Petzold et al., 2014). This supports the view that the genetic lineages represented by clade I and clade II should be treated as unconfirmed candidate species (Fritz et al., 2015), a category introduced by Padial et al. (2010) for groups of individuals within formally recognized species showing large genetic distances, but without further information. Unconfirmed candidate species deserve further study and additional characters may qualify them for the description as new species. Our present investigation revealed that lineages I and II also differ in one of the two nuclear markers (R35) to an extent resembling their divergence to *P. subrufa* sensu stricto. This supports that the two lineages represent distinct species and that a full taxonomic revision should be performed, involving morphological characters and resolving the nomenclatural issues described by Petzold et al. (2014). Within that work, sampling the putative contact zone between the westernmost records of lineage I and the easternmost records of lineage II will be mandatory. The two lineages are separated by a sampling gap of *circa* 220 km, corresponding to the region between the farms Chelance and Groenfontein near Worcester and Calitzdorp (Western Cape), respectively. The study of this contact zone and of other putative contact zones will provide insights in gene flow, possible hybridization and ongoing differentiation processes. Today, there is a broad array of mitochondrial and highly informative nuclear markers available, including SNPs, which are powerful tools for unravelling phylogeographic patterns and for understanding speciation processes beyond the description of the distribution of genetic lineages. Finally, the relatively small region revealed as potentially suitable for lineage II (Fig. 5) implies that the conservation of this candidate species requires more attention because surface water is scarce in this region.

## Conclusions

Nuclear markers confirm the distinctness of *Pelomedusa galeata* and *P. subrufa* sensu stricto. *Pelomedusa galeata* comprises two genetically deeply divergent lineages (I and II) that differ in mitochondrial and nuclear DNA. Lineage I shows considerable phylogeographic structure, with three distinct mitochondrial clades (Ia-Ic). *Pelomedusa galeata* and *P. subrufa*, as well as the four genetic clusters within *P. galeata* (Ia-Ic, II), seem to be distributed parapatrically, each occupying a distinct climatic niche. However, some niche overlap was found for *P. galeata* and *P. subrufa*. One lineage of *P. galeata* (II) appears to be largely confined to a small region in southwestern South Africa and deserves more conservational attention due to the scarcity of surface water there. Further studies should focus on putative contact zones of the two species and of the genetic clusters within *P. galeata* and use informative nuclear markers to examine gene flow and hybridization. In South Africa, records of *P. subrufa* are restricted to the northeast of the country (Mpumalanga, Limpopo). The species ranges approximately 350 km further westward than previously known and a sympatric occurrence with *P. galeata* is possible. Unlike *P. galeata*, no phylogeographic structure was found for *P. subrufa*, which seems to be related to historically stable versus fluctuating distribution ranges. Our investigation is one of the rare studies describing the phylogeography of sub-Saharan biota, by thus laying the foundation for unravelling general phylogeographic patterns. Such paradigms are likely to exist, but the lack of individual case studies impedes their identification. According to the little information available, it seems likely that physiographic barriers (e.g., the Great Escarpment) generally correlate with phylogeographic breaklines and contributed together with climatic factors to the establishment of phylogeographic structuring.

##  Supplemental Information

10.7717/peerj.4901/supp-1 Figs. S1–S7 Figures S1–S7

10.7717/peerj.4901/supp-2 Table S1 Studied samples and ENA accession numbers for DNA sequences

10.7717/peerj.4901/supp-3 Supplemental Information 1 Alignment 12S rRNA sequences

10.7717/peerj.4901/supp-4 Supplemental Information 2 Alignment cyt b sequences

10.7717/peerj.4901/supp-5 Supplemental Information 3 Alignment ND4 + tRNA sequences

10.7717/peerj.4901/supp-6 Supplemental Information 4 Alignment of concatenated mtDNA for TCS network

10.7717/peerj.4901/supp-7 Supplemental Information 5 Alignment ODC sequences

10.7717/peerj.4901/supp-8 Supplemental Information 6 Alignment R35 sequences

## Additional Information and Declarations

### Field Study Permissions

The following information was supplied relating to field study approvals (i.e., approving body and any reference numbers): Fieldwork and sampling in South Africa were permitted by Limpopo Provincial Government (permit ZA/Lp/80202), Ezemvelo KwaZulu-Natal Wildlife (permit OP 139/2017), CapeNature (permit AAA007-00212-0056), Department of Environmental Affairs, Eastern Cape (permit CRO117/13C & CRO 118/13CR), and Biodiversity Northern Cape Province (permit 245/2015). Fieldwork and sampling in Namibia were permitted by the Ministry of Environment and Tourism (permit 1910/2014).

### DNA Deposition

The following information was supplied regarding the deposition of DNA sequences: Sequences have been deposited at the European Nucleotide Archive (ENA) and accession numbers (LT992039–LT992252) are included in the Supplemental Information.
