# Supplementary material for: In quest of contact: phylogeography of helmeted terrapins (Pelomedusa galeata, P. subrufa sensu stricto)
Source: PeerJ. 2018 Jun 5;6:e4901. doi: 10.7717/peerj.4901 (PMC5993023; doi:10.7717/peerj.4901)
Supplement: Figs. S1–S7 [file peerj-06-4901-s001.pdf]

# In quest of contact: phylogeography of helmeted terrapins (*Pelomedusa galeata*, *P. subrufa* sensu stricto)

Melita Vamberger, Margaretha D. Hofmeyr, Flora Ihlow, Uwe Fritz

*Supplemental Information – PeerJ*

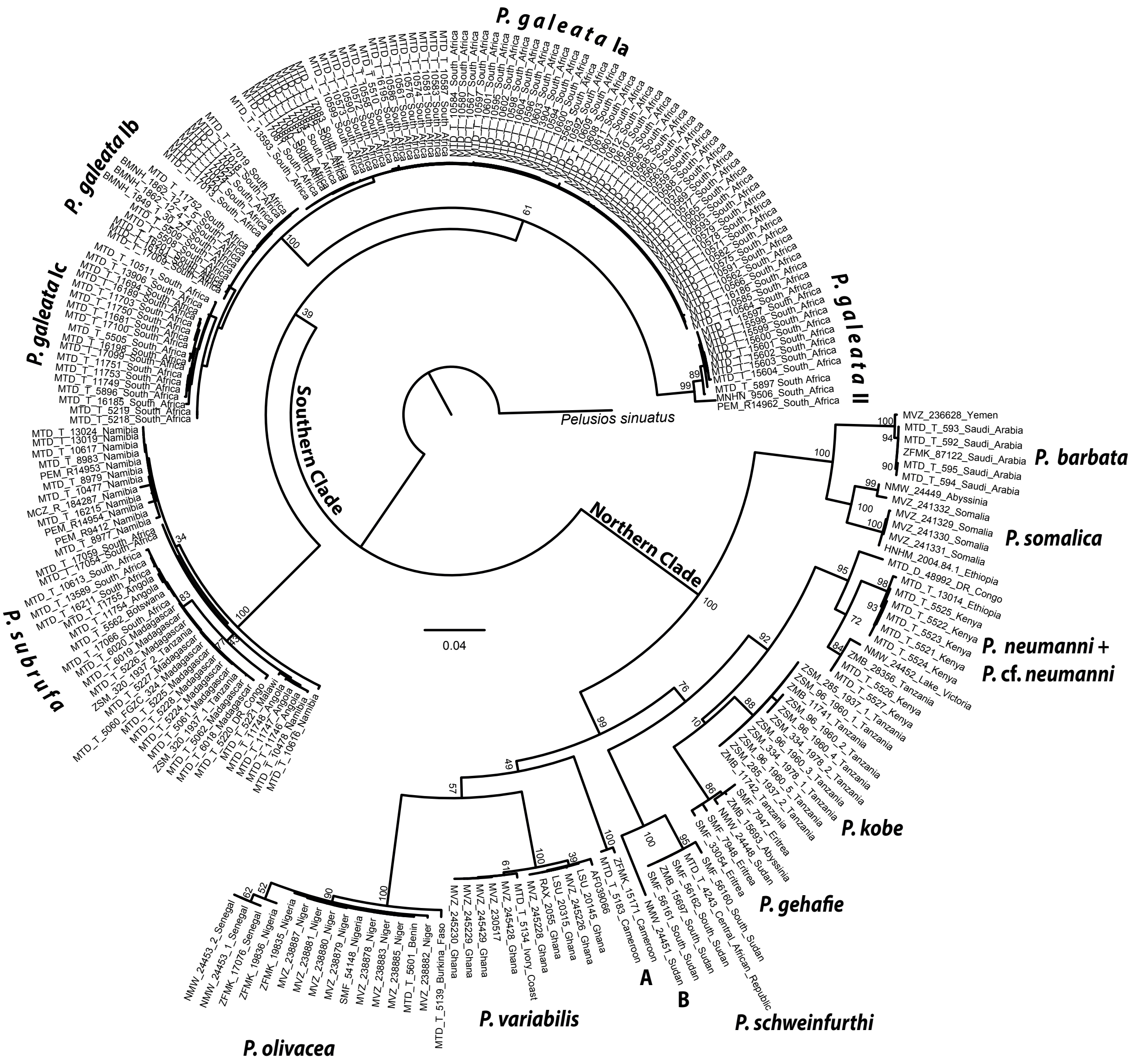


**Figure S1 Complete Maximum Likelihood tree, showing individual samples.** For sample codes not explained in Table S1, see Petzold et al. (2014) and Nagy et al. (2015).

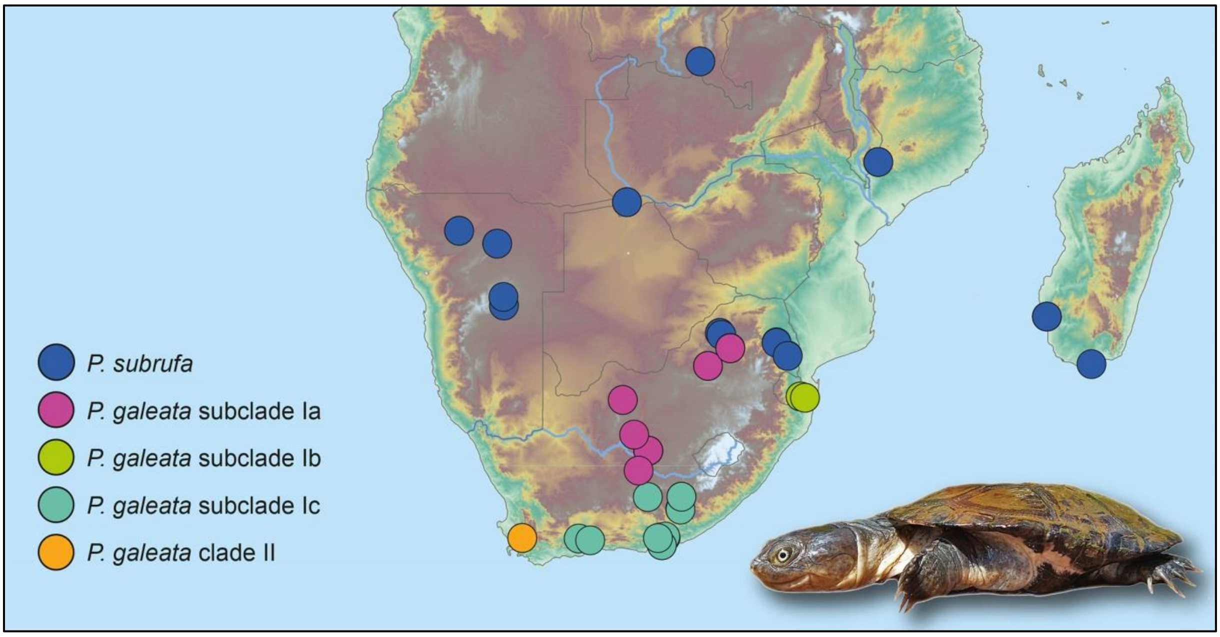


**Figure S2 Sampling sites with nuclear DNA information.** Color-coding corresponds to mitochondrial lineages and subclades.

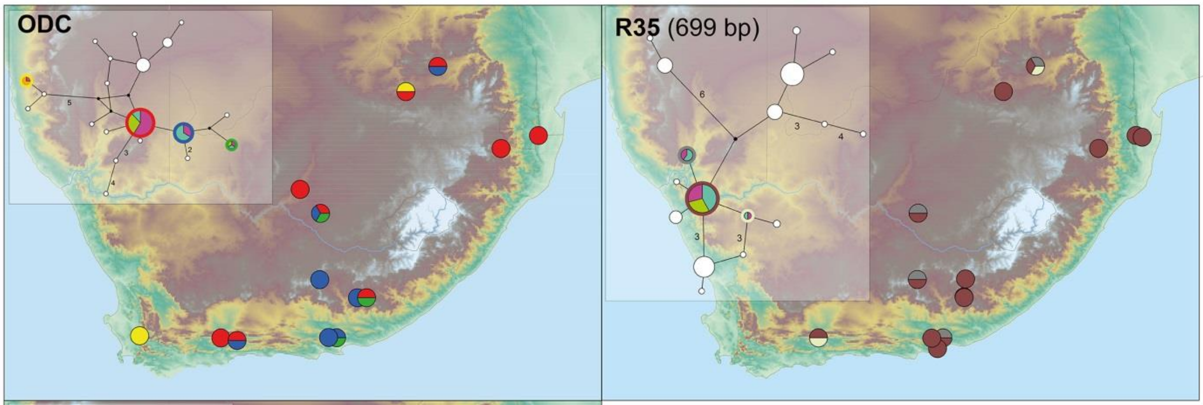


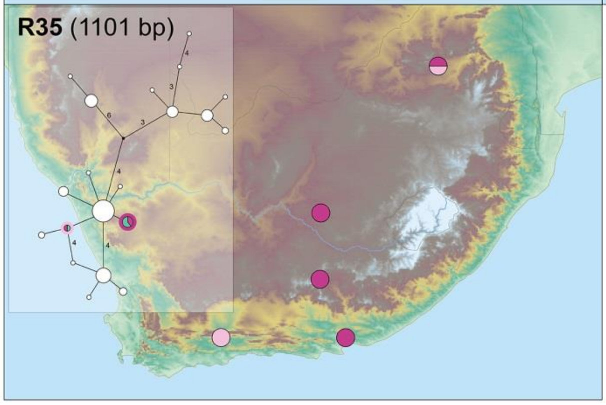


**Figure S3 Geographic distribution of shared nuclear haplotypes of lineages and subclades within *Pelomedusa galeata*.** Under gene flow, haplotype sharing should occur close to putative contact zones (cf. Fig. 2 for contact zones). However, shared haplotypes are distributed instead randomly, rather suggestive of ancestral polymorphism. Insets: haplotype networks (cf. Fig. 3), shared haplotypes color-coded.

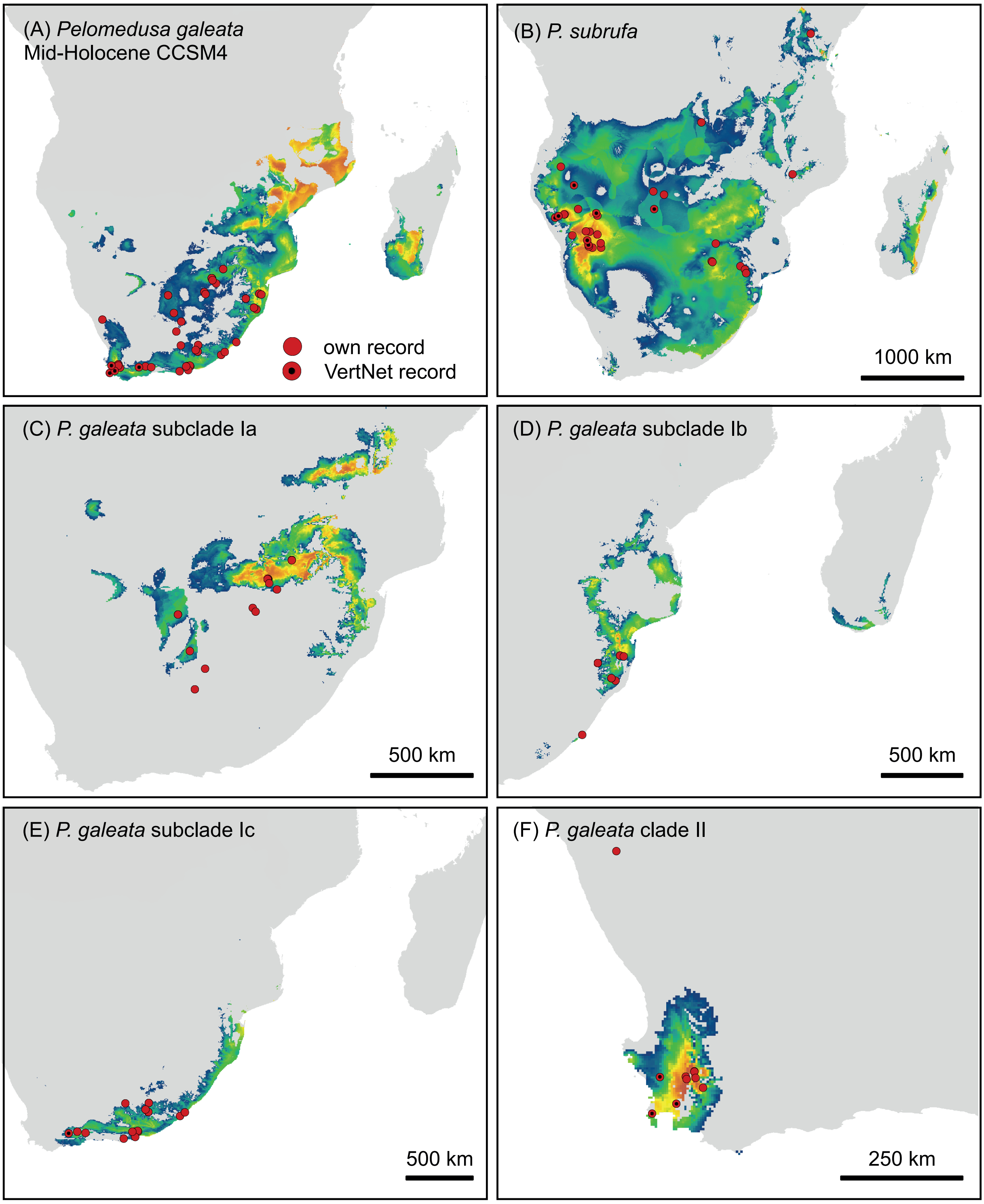


**Figure S4 Predicted climatic niches for *Pelomedusa galeata* (A), *P. subrufa* (B) and genetic clusters within *P. galeata* (C-F) during the mid-Holocene (CCSM4).** Probabilities for habitat suitability range from low (blue) to high (orange).

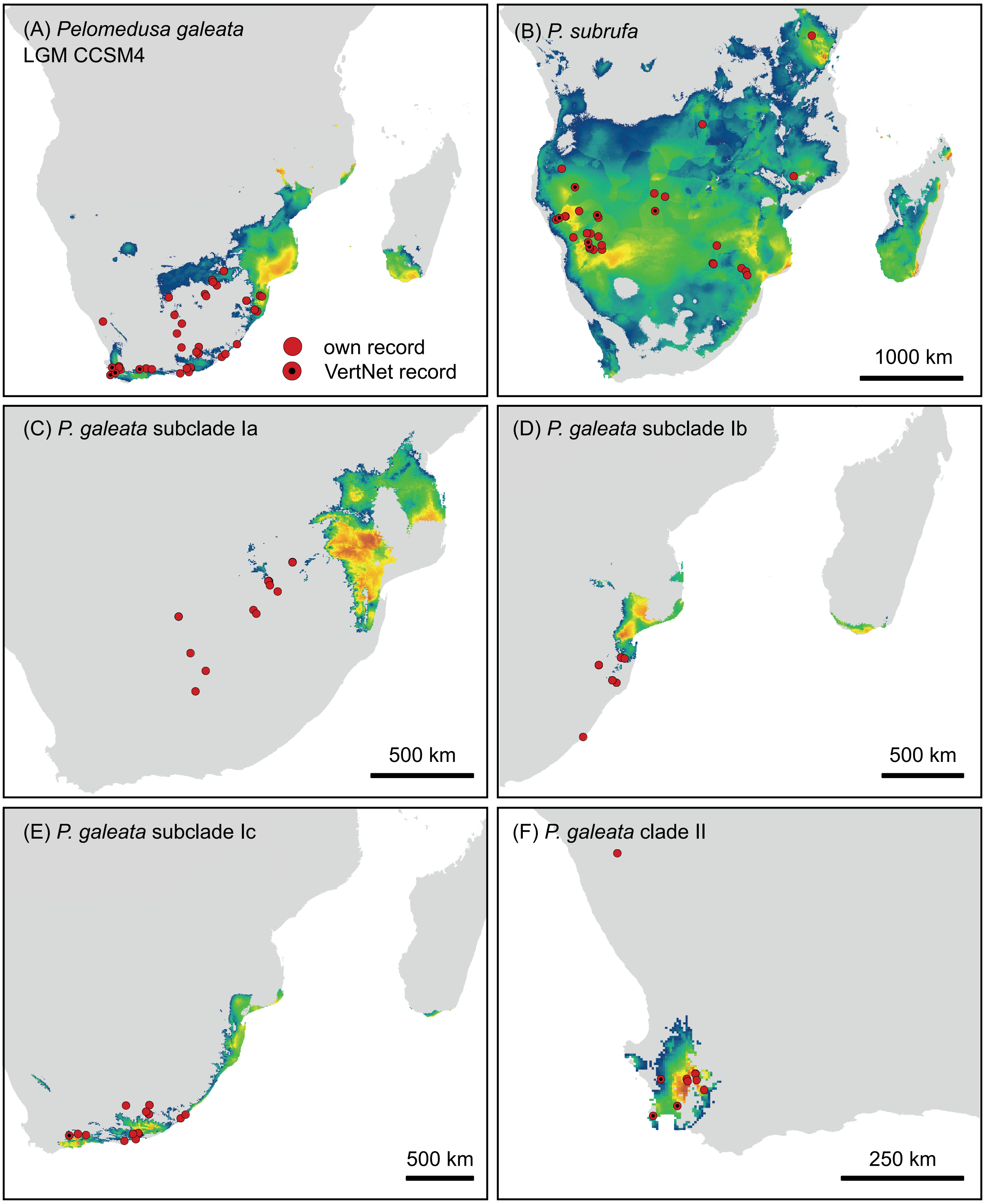


**Figure S5 Predicted climatic niches for *Pelomedusa galeata* (A), *P. subrufa* (B) and genetic clusters within *P. galeata* (C-F) during the Last Glacial Maximum (CCSM4).** Probabilities for habitat suitability range from low (blue) to high (orange).

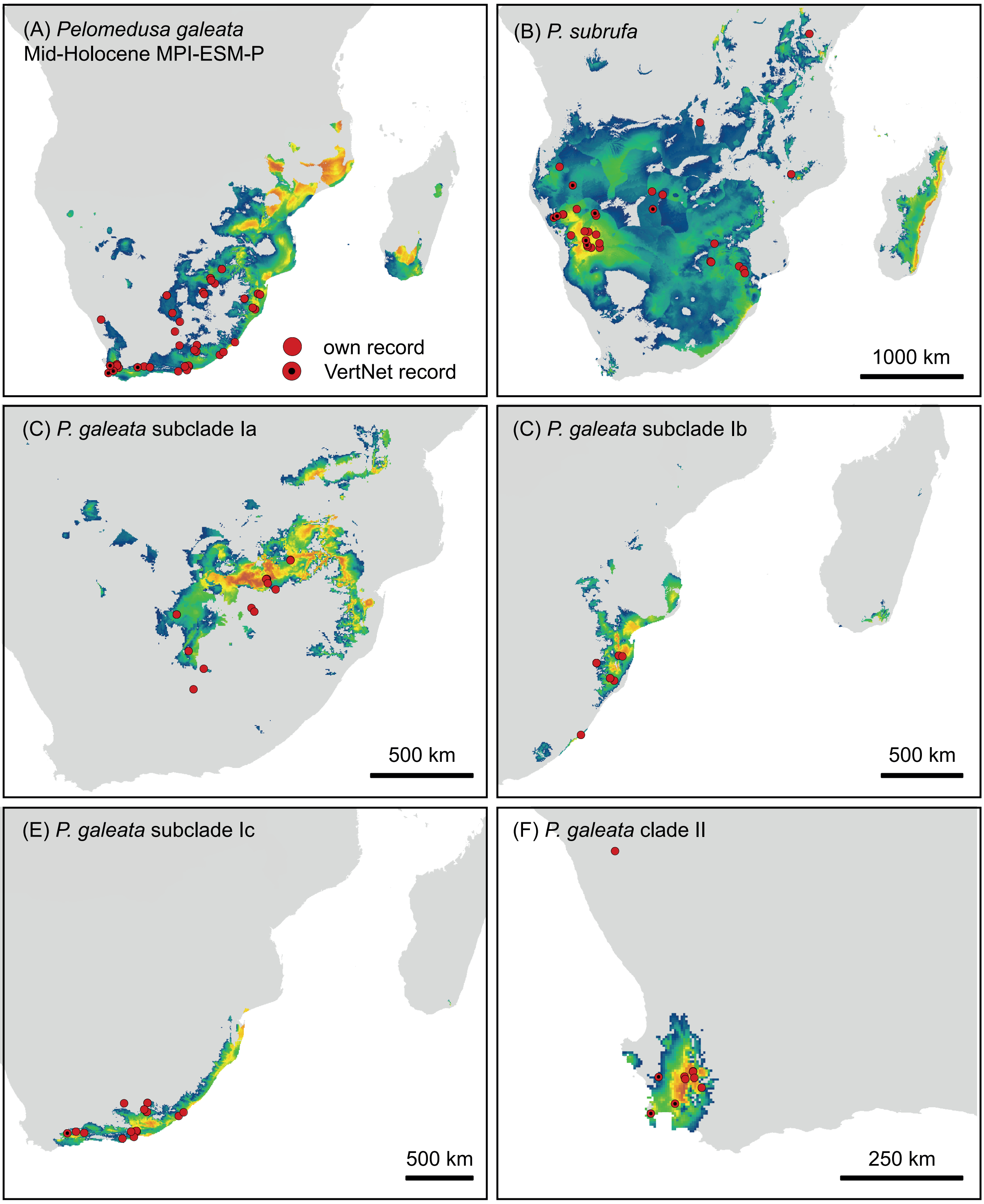


**Figure S6 Predicted climatic niches for *Pelomedusa galeata* (A), *P. subrufa* (B) and genetic clusters within *P. galeata* (C-F) during the mid-Holocene (MPI-ESM-P).** Probabilities for habitat suitability range from low (blue) to high (orange).

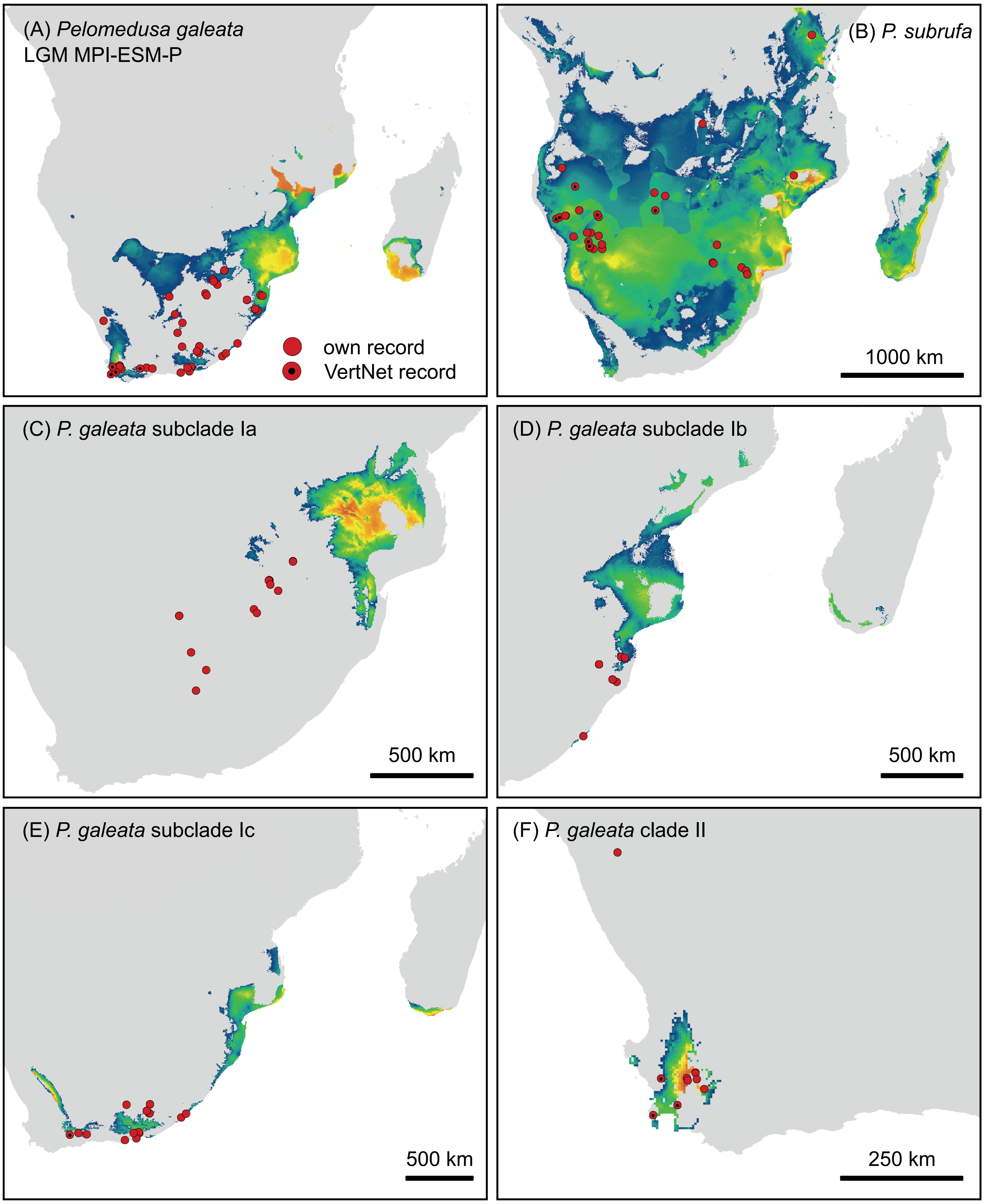


**Figure S7 Predicted climatic niches for *Pelomedusa galeata* (A), *P. subrufa* (B) and genetic clusters within *P. galeata* (C-F) during the Last Glacial Maximum (MPI-ESM-P).** Probabilities for habitat suitability range from low (blue) to high (orange).
